# Experimental data on the removal of acid orange 10 dye from aqueous solutions using TiO_2_/Na-Y zeolite and BiVO_4_/Na-Y zeolite nanostructures: A comparison study

**DOI:** 10.1016/j.dib.2021.106869

**Published:** 2021-02-12

**Authors:** Behzad Rahimi, Nayereh Rezaie-Rahimi, Negar Jafari, Ali Abdolahnejad, Afshin Ebrahimi

**Affiliations:** a Student Research Committee, School of Health, Isfahan University of Medical Sciences, Isfahan, Iran; b Department of Health, Safety and Environment, Pasteur Institute of Iran, Tehran, Iran; c Environment Research Center, Research Institute for Primordial Prevention of Non-communicable Disease, Isfahan University of Medical Sciences, Isfahan, Iran; d Department of Environmental Health Engineering, School of Health, Isfahan University of Medical Sciences, Isfahan, Iran; e Department of Public Health, Maragheh University of Medical Sciences, Maragheh, Iran

**Keywords:** Nanomaterials, TiO_2_/zeolite, BiVO_4_/zeolite, Acid orange 10, Dye degradation

## Abstract

The increase of textile factories, along with the continuous development of industrialization has led to excessive discharge of high toxicity wastewater along with a diverse range of contaminants in wastewater. In this regard, to reduce their operating costs and treatment time, in this work, two synthesized nanostructures, TiO_2_/Na-Y zeolite and BiVO_4_/Na-Y zeolite was compared to remove acid orange 10 (AO10) from the aqueous solutions. The obtained optimum operating conditions including initial dye concentration, initial pH, contact time, catalyst dosage and AO10 removal efficiency were 20 mg/L, 3, 7 min, 0.2 g/100 mL, and 99.77% for TiO_2_/Na-Y zeolite and 20 mg/L, 3, 200 min, 0.2 g/100 mL and 46.13% for BiVO_4_/Na-Y zeolite composite, respectively. The structural characteristics of the synthetized materials were also determined by X-ray diffraction (XRD), field emission scanning electron microscopy (FESEM), and fourier-transform infrared spectroscopy (FTIR).

## Specifications Table

**Table:** 

Subject	Environmental Chemistry
Specific subject area	Adsorption
Type of data	Table, image, and figure
How data was acquired	The initial and final AO10 concentration was analyzed by measuring its maximum absorbance (λ_max_ = 475 nm) using a DR-5000, HACH LANGE, USA spectrophotometer. The crystal structure analysis of the nanomaterials was detected via XRD device. The morphology observation was also detected by a field-emission scanning electron microscope (FE-SEM, the MIRA3 model, developed by TESCAN Company). Fourier transform infrared spectra (FTIR) was analyzed by Tensor 27-Equinox 55 model, Bruker corporation. RSM was employed to evaluate the main interaction effects and to optimize the number of nanomaterial process experiments using the Design-Expert 11.0.1 software.
Data format	Raw, Analyzed
Parameters for data collection	XRD device, Bruker Corporation (Germany) using a lamp Cu Kα with a wavelength equal to 1.7890^°^A at 40 kV and 40 mA, in the range of 2θ = (10 to 90)^°^. A field-emission scanning electron microscope (FESEM), the MIRA3 model, developed by TESCAN company, operating voltage = 15 kV. The fourier transform infrared spectroscopy (FTIR) was in the range of 400–4000 cm^−1^ with a resolution of 4 cm^−1^.
Description of data collection	Degradation of acid orange 10 by TiO_2_/Na-Y zeolite and BiVO_4_/Na-Y zeolite composite
Data source location	Isfahan University of Medical Sciences, Isfahan, Iran
Data accessibility	Data are available in this article
Related research article	A. Ebrahimi, N. Jafari, K. Ebrahimpour, A. Nikoonahad, A. Mohammadi, F. Fanaei, A. Abdolahnejad The performance of TiO_2_/NaY-zeolite nanocomposite in photocatalytic degradation of Microcystin-LR from aqueous solutions: Optimization by response surface methodology (RSM) Environmental Health Engineering and Management Journal http://10.34172/EHEM.2020.29

## Value of the Data

• TiO_2_/zeolite and BiVO_4_/zeolite composites which were synthesized by hydrothermal method, would be useful for the removal of toxic pollutants such as acid orange 10(AO10) dye from water and wastewater.
• These data show the better removal efficacy of TiO_2_/zeolite composite compared to BiVO_4_/zeolite composite on acid orange 10 removal.
• Process optimization using response surface methodology (RSM) by TiO_2_/zeolite and BiVO_4_/zeolite composites for dye removal yielded 99.77% and 46.13%, respectively.

## 1 Data Description

**Fig. 1:**
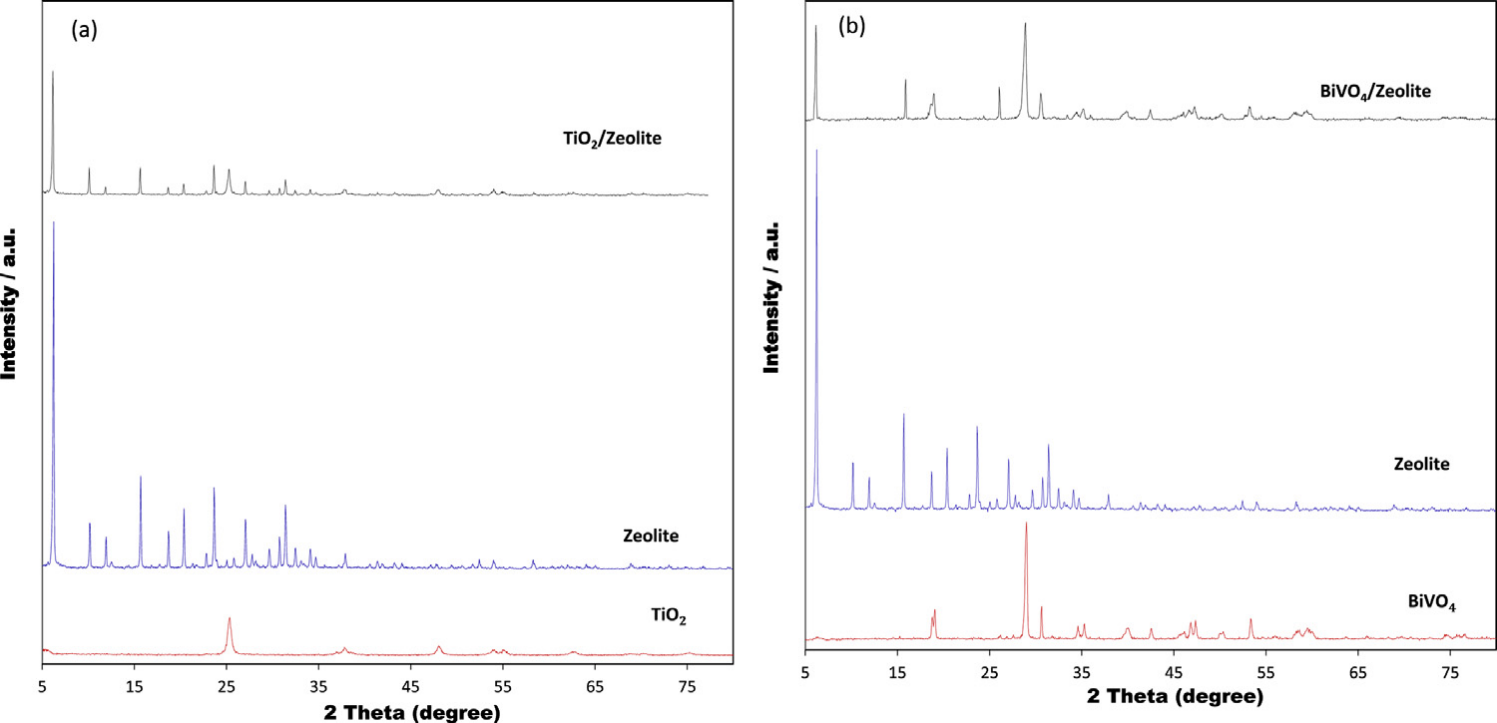
The XRD pattern of the studied materials: a) TiO_2_/Zeolite, TiO_2_, Zeolite;b) BiVO_4_/Zeolite, BiVO_4_, Zeolite.

**Fig. 2:**
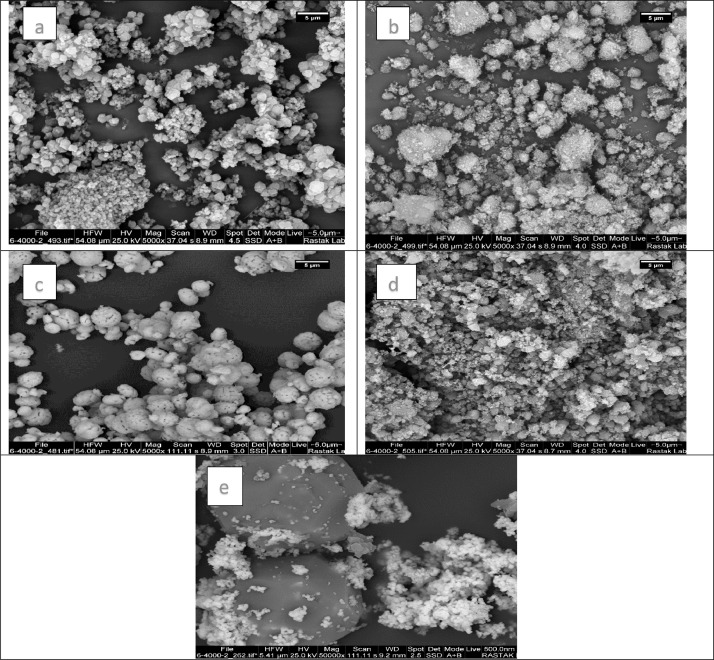
The FE-SEM images of the prepared nanomaterials of a) Zeolite, b) TiO_2_, c) BiVO_4_, d) TiO_2_/zeolite, e) BiVO_4_/zeolite.

**Fig. 3:**
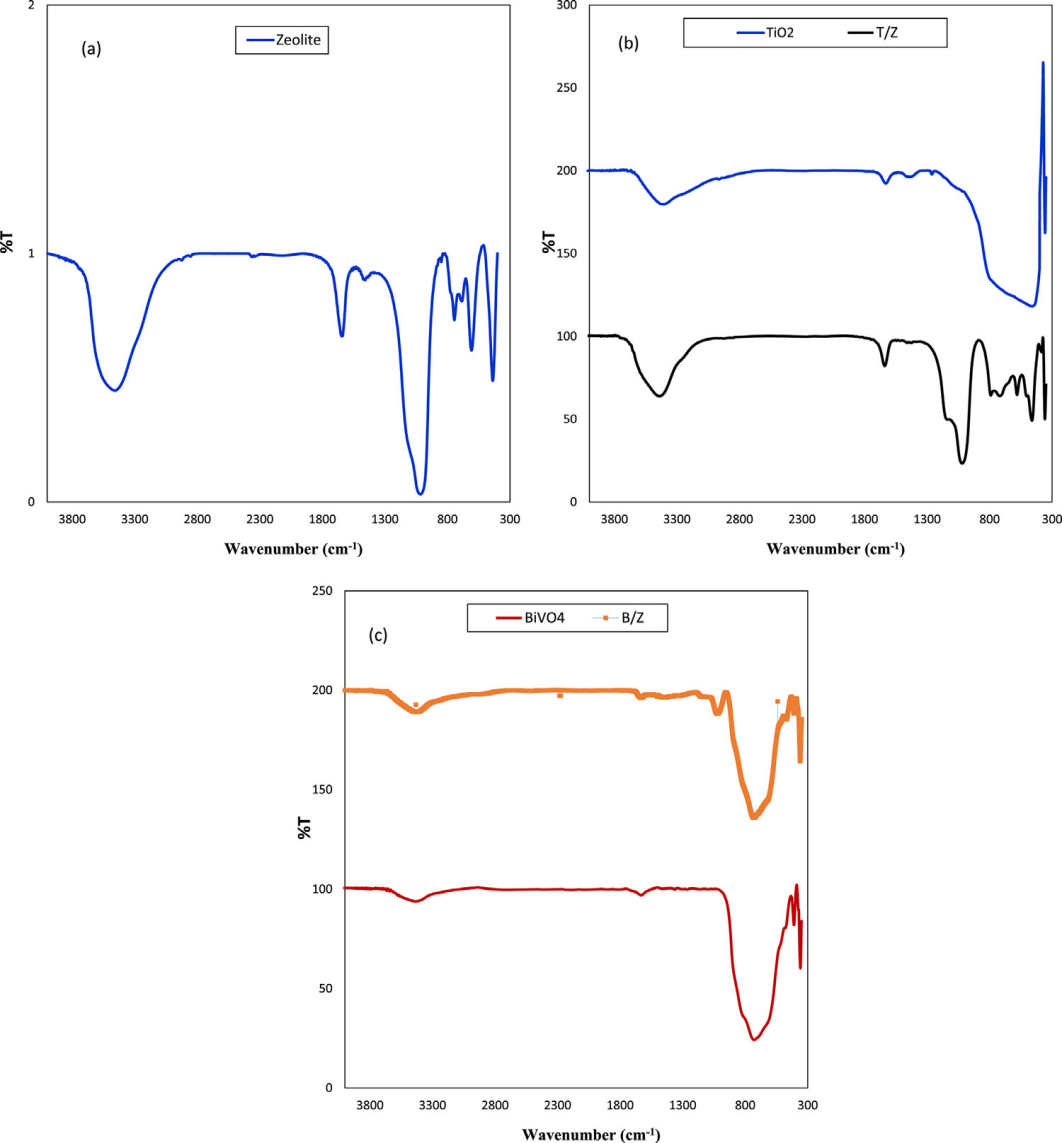
The FT-IR pattern of the studied materials of a) Zeolite b) TiO_2_/Zeolite, TiO_2_ c) BiVO_4_/Zeolite, BiVO_4_.

**Fig. 4:**
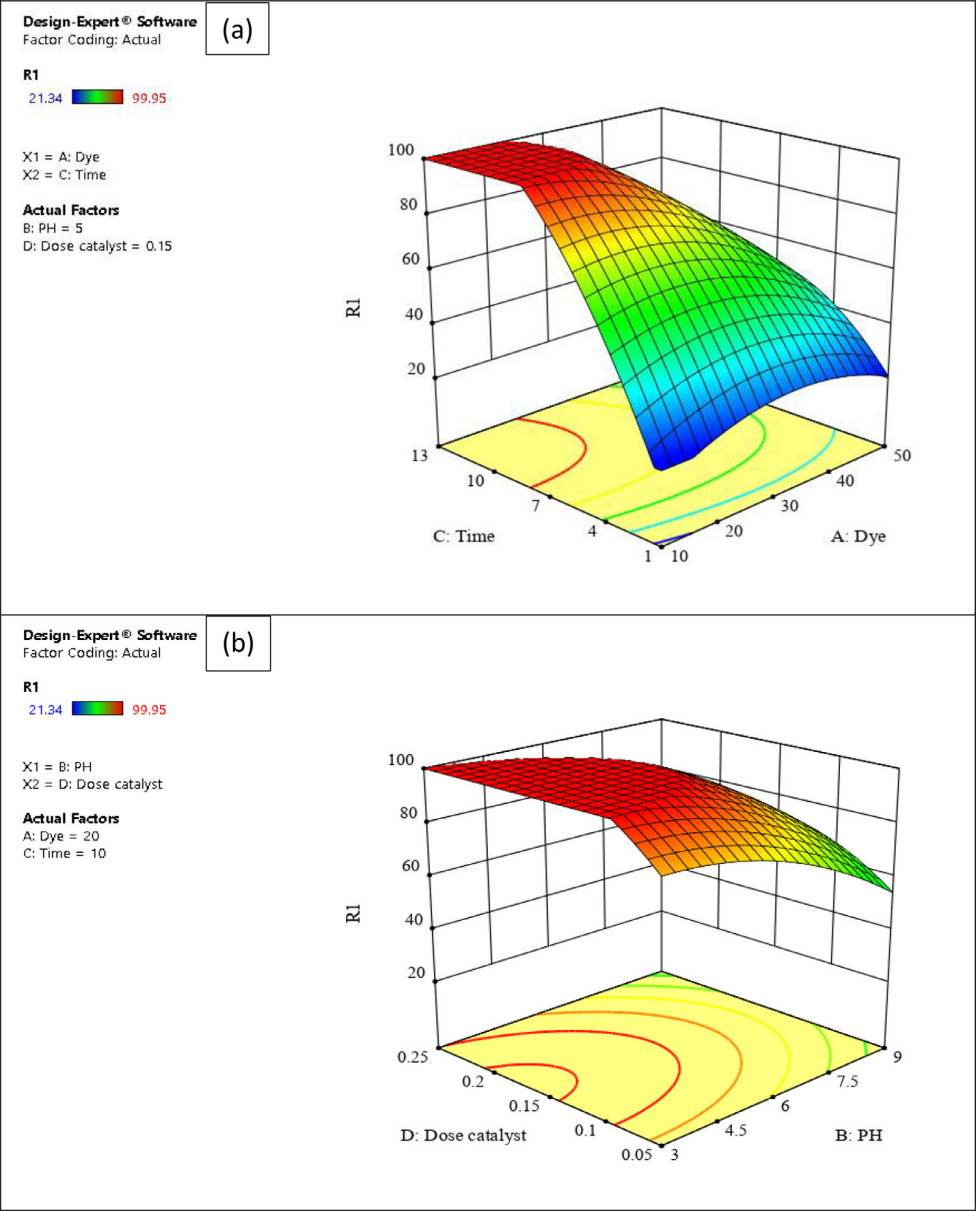
Results of 3-D surface plots on AO10 removal efficiency for TiO_2_ /zeolite composite of a) Time and Dye b) Dose catalyst and pH.

**Fig. 5:**
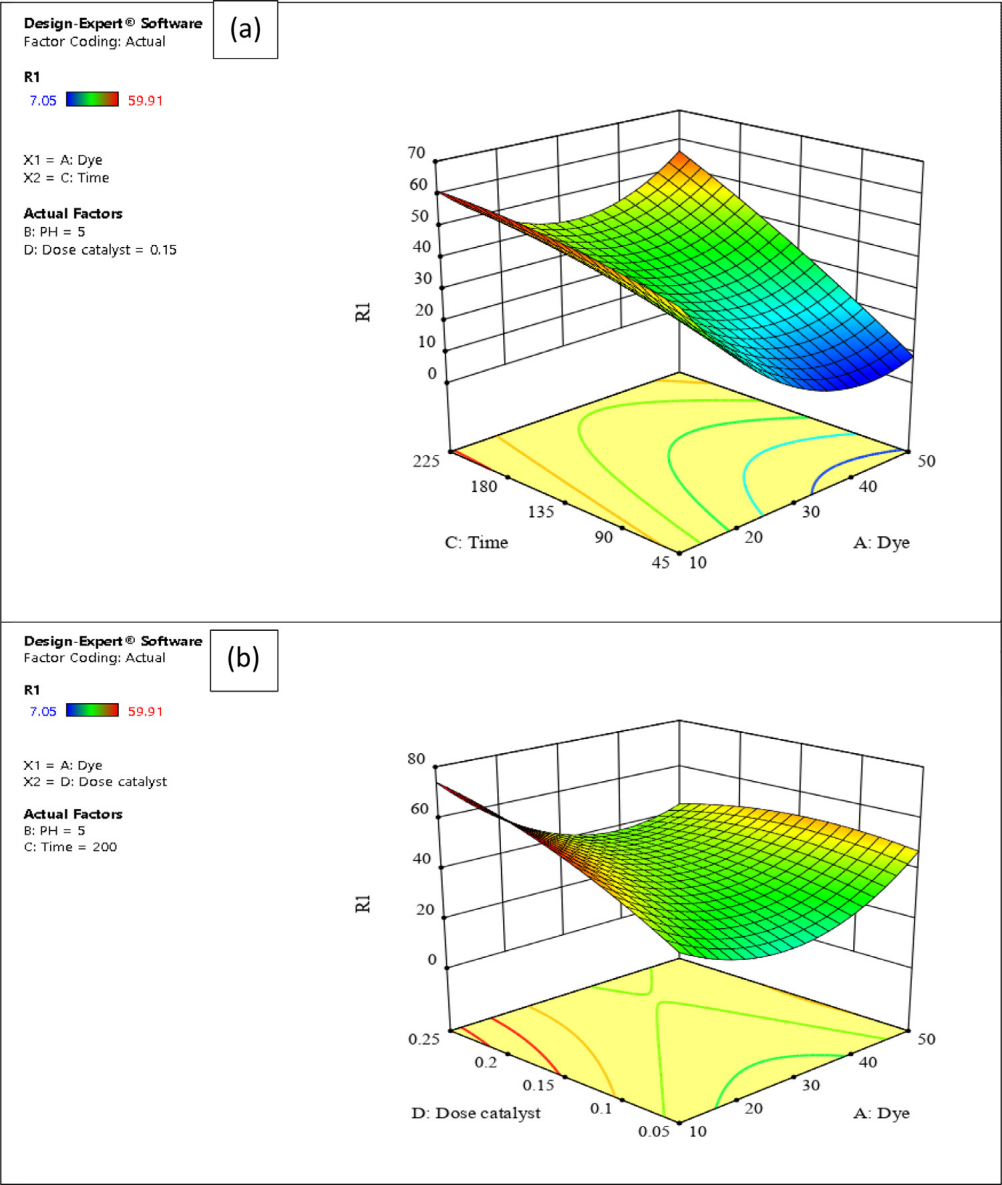
Results of 3-D surface plots on AO10 removal efficiency for BiVO_4_/zeolite composite of a) Time and Dye b) Dose catalyst and Dye.

**Fig. 6:**
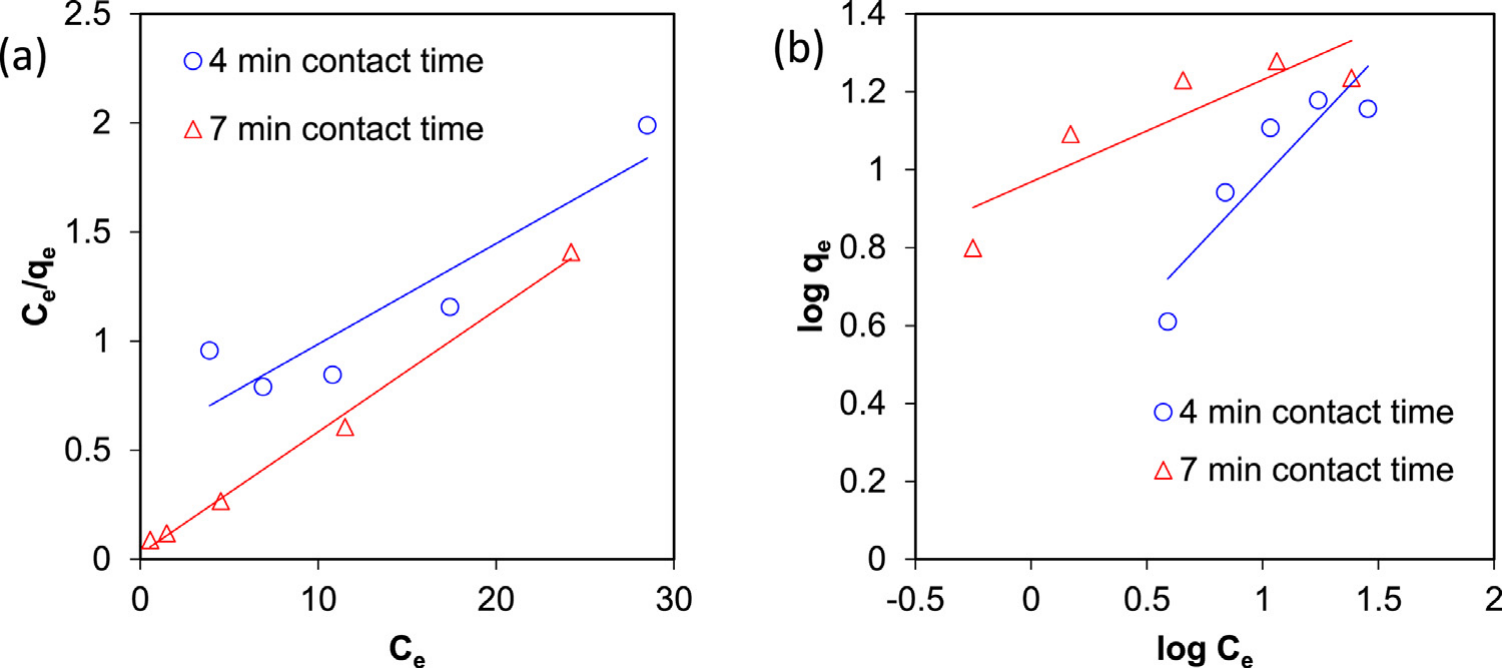
Adsorption isotherms for AO10 removal on TiO_2_/zeolite composite of a) Langmuir and b) Freundlich.

**Fig. 7:**
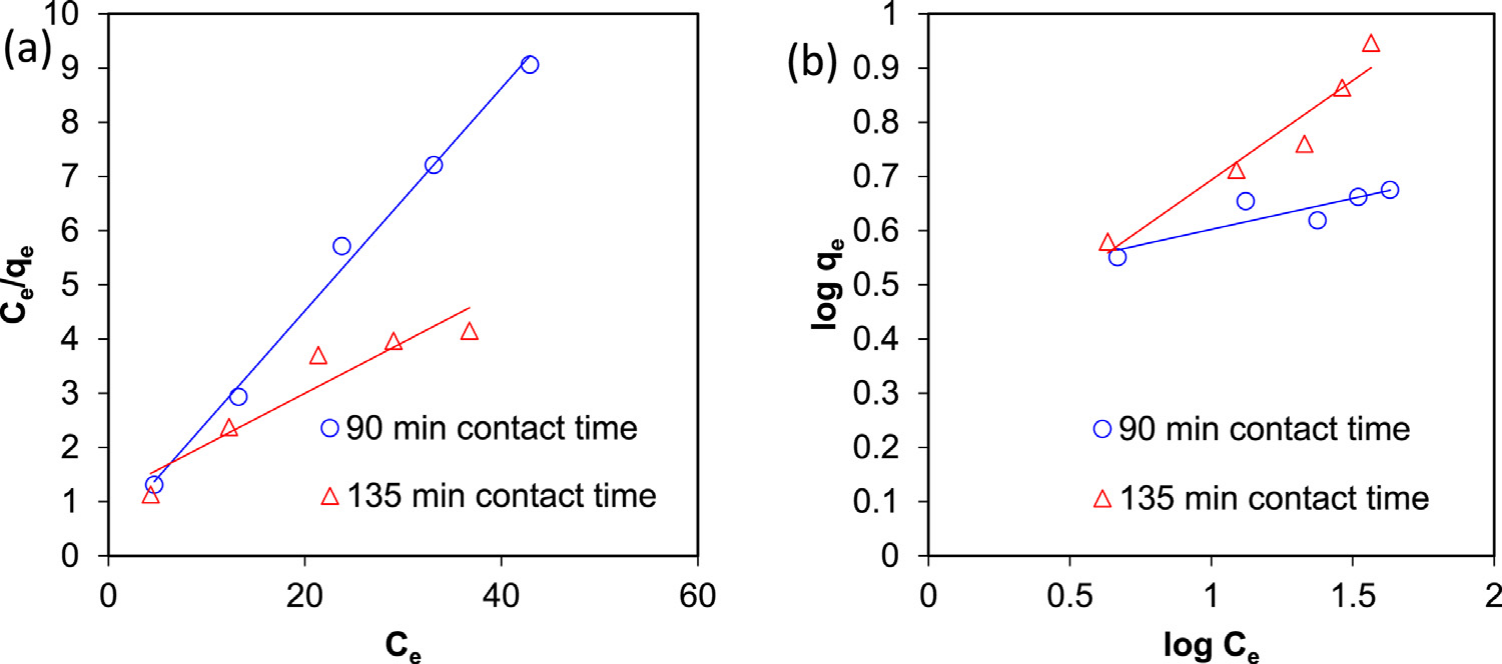
Adsorption isotherms for AO10 removal on BiVO_4_/zeolite composite of a) Langmuir and b) Freundlich.

**Fig. 8:**
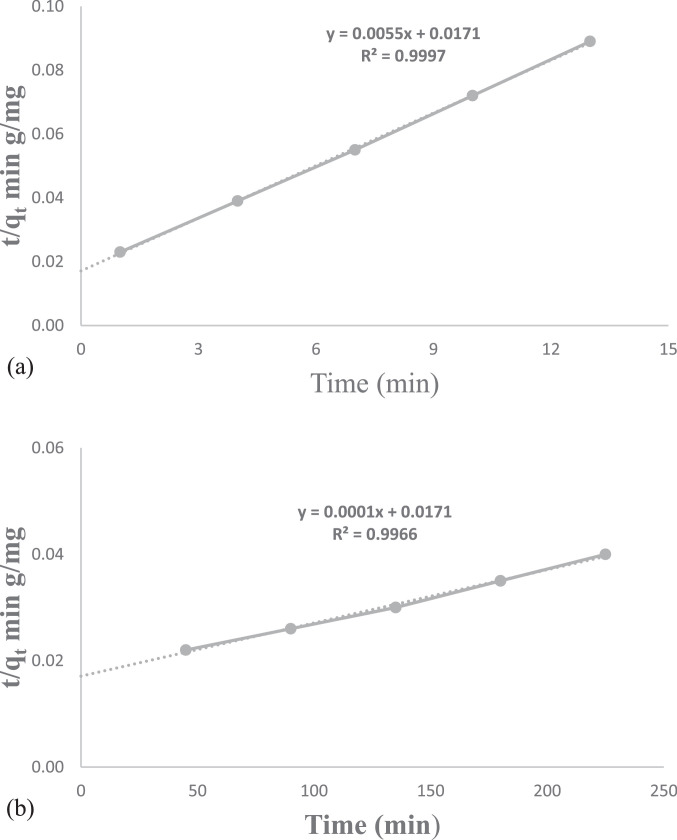
Pseudo-second order model for nanostructure on AO10 dye removal: a) TiO_2_/zeolite and b) BiVO_4_/zeolite.

**Fig. 9:**
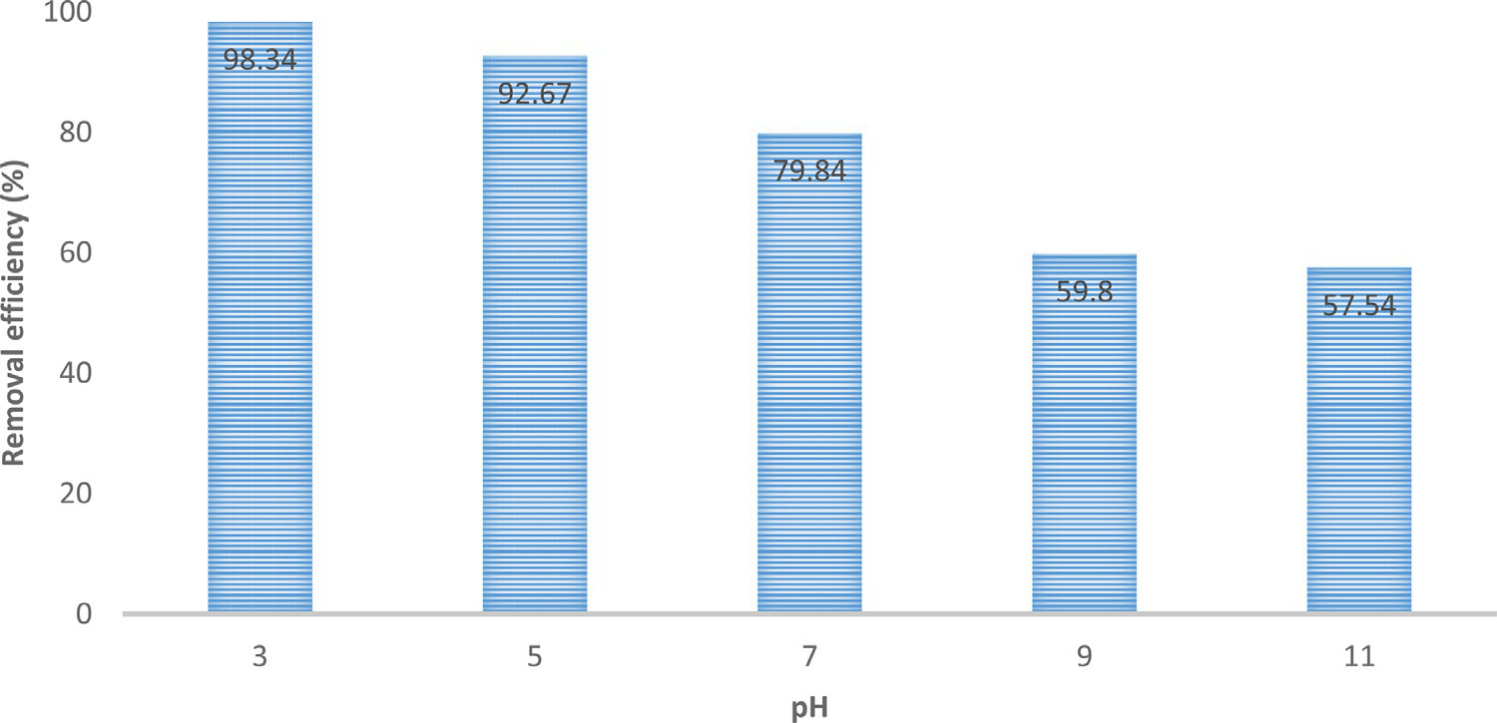
Effect of pH on AO10 dye removal by TiO_2_/zeolite nanostructure (dye concentration = 20 mg/L, contact time =  7 min, catalyst dosage =  0.2 g/100 mL).

**Fig. 10:**
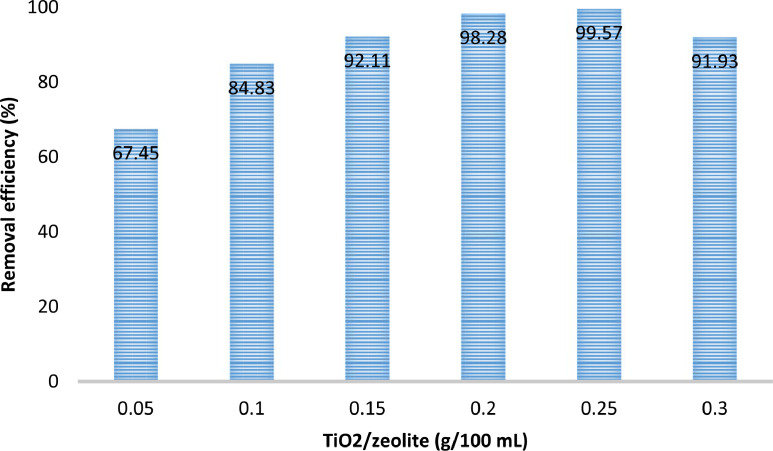
Effect of TiO_2_/zeolite dosage on AO10 dye removal (dye concentration = 20 mg/L, pH = 3, contact time = 7 min,).

**Fig. 11:**
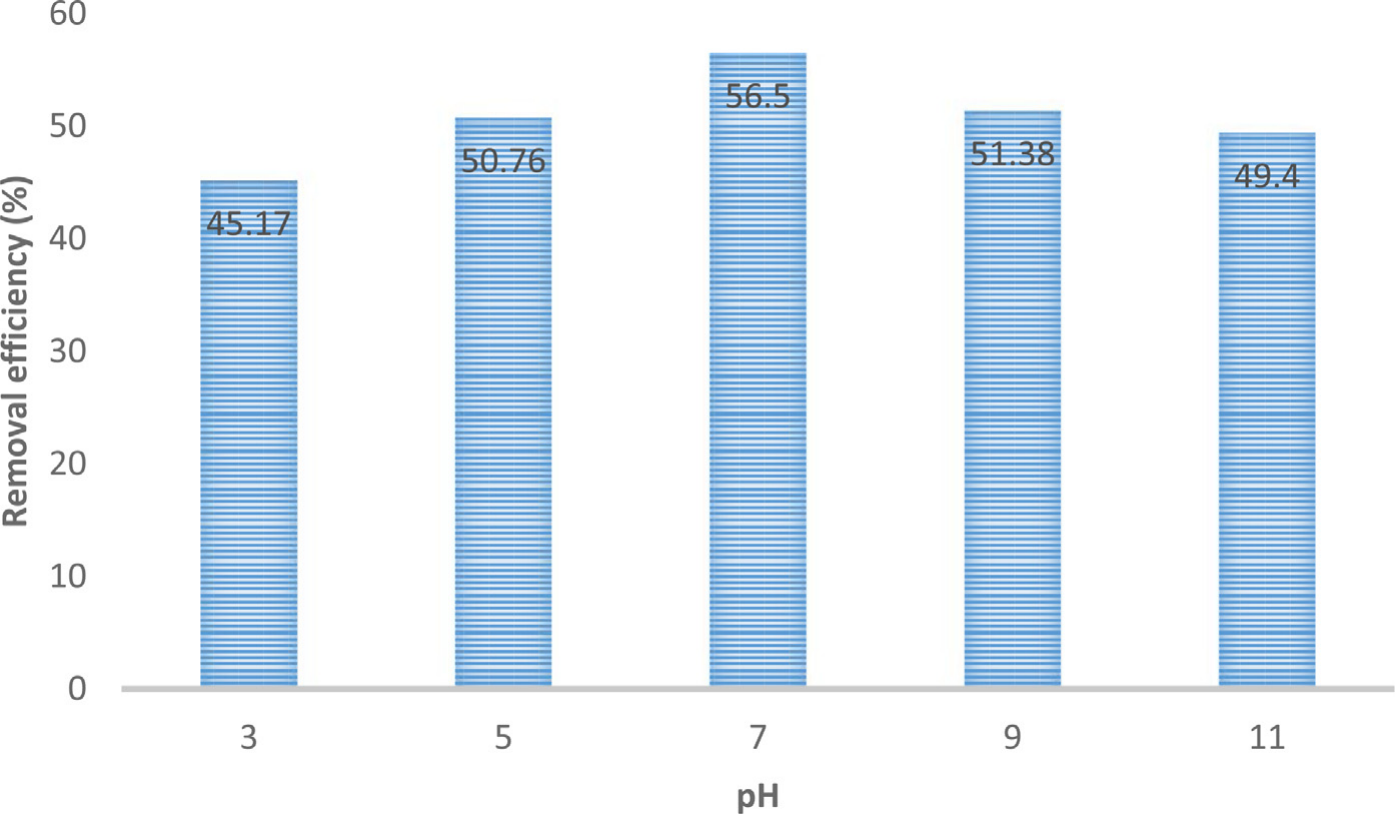
Effect of pH on AO10 dye removal by BiVO_4_/zeolite nanostructure (dye concentration = 20 mg/L, Contact time = 200 min, catalyst dosage =  0.2 g/100 mL).

**Fig. 12:**
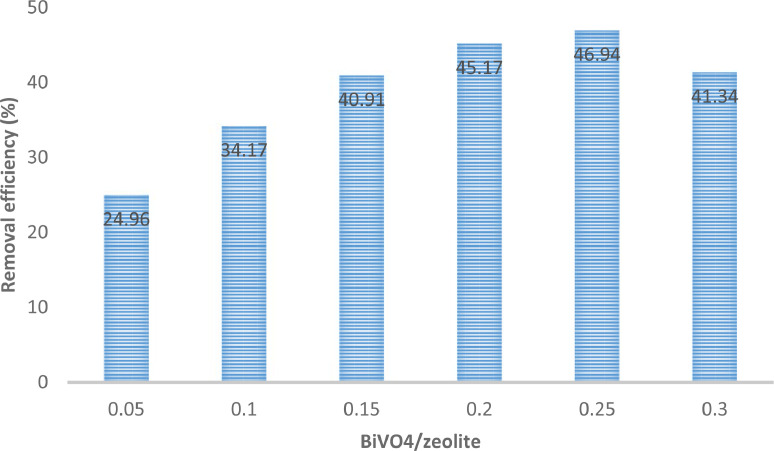
Effect of BiVO_4_/zeolite dosage on AO10 dye removal (dye concentration = 20 mg/L, pH = 3, Contact time =  200 min).

The presented data described the removal of acid orange 10 (AO10) dye by TiO_2_/zeolite and BiVO_4_/zeolite composites. The XRD pattern of TiO_2_/zeolite, TiO_2_, zeolite, BiVO_4_/zeolite, and BiVO_4_ are presented in Fig. 1. The sharp peaks of anatase TiO_2_ are placed at 2θ of 25.35^°^, 37.8^°^, 48.05^°^, 54.95^°^, 55.05^°^, and 62.55 and for BiVO_4_ are displayed at 2θ of 19.05^°^, 29^°^, 35.3^°^, and 47.35^°^indicating successful synthesis of BiVO_4_ nanoparticle. Also, all the identified dominant peaks at 2θ belong to different minerals including Na, Al, and Si present in the XRD pattern of Na-Y zeolite with Na_5_Al_6_Si_30_O_72_ .18H_2_O formula. Fig. 1 also demonstrates the presence of Ti and Bi phase in Na-Y zeolite structure, while in the XRD pattern of Na-Y zeolite, these phases are not observed, indicating the successful coupling of TiO_2_ and BiVO_4_ to Na-Y zeolite structure. These results indicate that bismuth vanadate and titanium dioxide were not destroyed during the synthesis preparation process. The FE-SEM images of nanomaterials are illustrated in Fig. 2. The FTIR pattern of the studied materials is also is represented in Fig. 3. Figs. 4 and 5 show the results of various factors in the form of three-dimensional surface plots on AO10 degradation efficiency for TiO_2_/zeolite and BiVO_4_/zeolite, respectively. Adsorption isotherms for AO10 removal on TiO_2_/zeolite and BiVO_4_/zeolite composite are presented in Figs. 6 and 7, respectively. Pseudo-second order kinetics for AO10 dye removal by TiO_2_/zeolite and BiVO_4_/zeolite are shown in Fig. 8. Figs. 9–12 illustrate the effects of nanomaterial dosage and pH on dye removal by the two studied nanostructures.

Specification of the AO10 is presented in Table 1. The properties of the Na-Y zeolite technical sheet are represented in Table 2. Tables 3 and 4 illustrate studied variables and ranges for AO10 dye removal by TiO_2_/zeolite and BiVO_4_/zeolite, respectively, based on the Design-Expert 11.0.1 software. Design response level experiments based on the coded values for dye degradation are listed in Table 5. Results of analysis of variance (ANOVA) for AO10 removal efficiency model by TiO_2_/zeolite and BiVO_4_/zeolite are shown in Tables 6 and 7, respectively. Parameter values of Langmuir and Freundlich isotherm results also are represented in Table 8. Correlation coefficients of pseudo-first and second order kinetic models are shown in Table 9.

**Table 1:** Chemical properties of the AO10.

Chemical name	Acid Orange 10
Molecular weight (g/mol)	452.36
Maximum wavelength	475 nm
Structure	
Formula	C_16_H_10_N_2_Na_2_O_7_S_2_

**Table 2:** Specifications of Na-Y zeolite technical sheet.

Cation	Na
Purity (%wt.)	≥99
Na_2_O (%wt.)	12
Form	white powder
Shape	Sphere
Pore size (A°)	7.5
Average particle size (μm)	0.5–1
SiO_2_/Al_2_O_3_ (mol/mol)	6
BET surface area (m^2^/g)	700
Bulk density (g/mL)	0.65
Water content in package (%wt.)	≤2
Pore volume (mL/g)	0.2
X-ray crystallography (%)	95
Lattice constant (A°)	24.39
Ignition loss (550 °C, 3 h) (%wt.)	8

**Table 3:** Studied variables and ranges for AO10 dye removal by TiO_2_/Zeolite composite.

	Ranges and levels				
Independent variables	−2	−1	0	+1	+2
Initial dye concentration (mg/L) (A)	10	20	30	40	50
Initial pH (B)	3	4.5	6	7.5	9
Contact time (min) (C)	1	4	7	10	13
Catalyst dosage (g/100 mL) (D)	0.05	0.1	0.15	0.2	0.25

**Table 4:** Studied variables and ranges for AO10 dye removal by BiVO_4_/Zeolite composite.

	Ranges and levels				
Independent variables	−2	−1	0	+1	+2
Initial dye concentration (mg/L) (A)	10	20	30	40	50
Initial pH (B)	3	4.5	6	7.5	9
Contact time (min) (C)	45	90	135	180	225
Catalyst dosage (g/100 mL) (D)	0.05	0.1	0.15	0.2	0.25

**Table 5:** Design response level experiments based on coded values for dye removal.

						Removal efficiency (%)			
						TiO_2_/zeolite		BiVO_4_/zeolite	
Std	Run	A	B	C	D	Exp.	Pred.	Exp.	Pred.
8	1	1	1	1	−1	51.52	57.88	29.66	30.62
23	2	0	0	0	−2	64.87	59.92	13.94	14.97
21	3	0	0	−2	0	21.34	25.84	17.76	15.65
9	4	−1	−1	−1	1	71.09	69.52	35.76	37.58
14	5	1	−1	1	1	65.99	66.10	34.71	36.47
24	6	0	0	0	2	67.43	64.61	34.98	31.19
10	7	1	−1	−1	1	55.03	54.14	15.68	17.45
20	8	0	2	0	0	60.93	56.39	21.72	21.90
4	9	1	1	−1	−1	45.97	40.55	14.05	15.97
3	10	−1	1	−1	−1	36.44	41.11	18.09	19.11
12	11	1	1	−1	1	41.84	39.69	15.37	21.97
17	12	−2	0	0	0	99.95	88.86	59.91	57.14
2	13	1	−1	−1	−1	51.05	51.69	15.89	17.10
18	14	2	0	0	0	44.91	48.23	31.82	33.33
15	15	−1	1	1	1	81.23	85.37	50.13	51.70
7	16	−1	1	1	−1	79.25	83.13	37.59	35.31
22	17	0	0	2	0	92.08	79.81	41.93	43.27
29	18	0	0	0	0	76.77	80.47	27.87	27.78
13	19	−1	−1	1	1	97.76	100	46.95	47.84
26	20	0	0	0	0	77.61	80.47	25.89	27.78
1	21	−1	−1	−1	−1	54.93	55.49	25.75	27.01
27	22	0	0	0	0	78.51	80.47	28.39	27.78
5	23	−1	−1	1	−1	93.68	100	34.08	35.33
6	24	1	−1	1	−1	72.07	72.12	36.97	34.18
11	25	−1	1	−1	1	48.89	51.83	31.28	33.56
25	26	0	0	0	0	79.91	80.47	25.71	27.78
30	27	0	0	0	0	82.98	80.47	27.36	27.78
16	28	1	1	1	1	46.11	48.54	38.54	36.78
19	29	0	−2	0	0	91.55	88.33	31.93	29.48
28	30	0	0	0	0	87.06	80.47	31.45	27.78

**Table 6:** Results of analysis of variance (ANOVA) for AO10 dye removal efficiency model by TiO_2_/zeolite.

Source	Sum of squares	df	Mean square	*F* value	*P*-value Prob > F
Model	10994.94	14	785.35	16.60	< 0.0001
A	2475.99	1	2475.99	52.33	< 0.0001
B	1529.45	1	1529.45	33.33	< 0.0001
C	4369.95	1	4369.95	92.35	< 0.0001
D	33.02	1	33.02	0.69	0.4166
AB	10.48	1	10.48	0.22	0.6447
AC	609.72	1	609.72	12.89	0.0027
AD	134.04	1	134.04	2.83	0.1131
BC	9.66	1	9.66	0.20	0.6579
BD	10.97	1	10.97	0.23	0.6371
CD	71.78	1	71.78	1.52	0.2370
A^2^	243.97	1	243.97	5.16	0.0383
B^2^	113.02	1	113.02	2.39	0.1431
C^2^	1310.57	1	1310.57	27.70	< 0.0001
D^2^	568.44	1	568.44	12.01	0.0035

Lack of fit: 0.063; R^2^: 0.93; Adeq precision: 16.5; Std. Dev.: 6.88.

**Table 7:** Results of analysis of variance (ANOVA) for AO10 dye removal efficiency model by BiVO_4_/zeolite.

Source	Sum of squares	df	Mean square	*F* value	*P*-value Prob > F
Model	4194.40	14	299.60	40.04	< 0.0001
A	924.30	1	924.30	123.52	< 0.0001
B	87.26	1	87.26	11.53	0.0040
C	1859.97	1	1859.97	248.56	< 0.0001
D	420.17	1	420.17	56.15	< 0.0001
AB	12.57	1	12.57	1.68	0.2146
AC	76.65	1	76.65	10.24	0.0060
AD	104.45	1	104.45	13.96	0.0020
BC	62.02	1	62.02	8.29	0.0115
BD	15.05	1	15.05	2.01	0.1765
CD	3.72	1	3.72	0.49	0.4913
A^2^	492.47	1	492.47	65.81	< 0.0001
B^2^	7.49	1	7.49	1.00	0.3328
C^2^	7.67	1	7.67	1.03	0.3373
D^2^	42.10	1	42.10	5.63	0.0315

Lack of fit: 0.2192; R^2^: 0.97; Adeq precision: 26.61; Std. Dev.: 2.74.

**Table 8:** Constant values of Langmuir and Freundlich isotherm results.

			Contact time (min)	
Type of composite	Isotherm model	Constant	4	7
TiO_2_/zeolite	Langmuir	K_L_	34.92	696.53
		Q_m_	21.69	17.90
		R^2^	0.84	0.99
	Freundlich	K_f_	1.05	0.031
		1/n	0.63	0.26
		R^2^	0.81	0.77
BiVO_4_/zeolite	Langmuir	K_L_	11.77	8.53
		Q_m_	4.87	10.60
		R^2^	0.99	0.89
	Freundlich	K_f_	0.71	1.11
		1/n	0.11	0.36
		R^2^	0.76	0.92

**Table 9:** Kinetic parameters for the adsorption of AO10 dye on TiO_2_/zeolite and BiVO_4_/zeolite.

Type of composite	Kinetic models	Constant	Values
TiO_2_/zeolite	Pseudo first-order	K_1_	0.107
		R^2^	0.84
	Pseudo second-order	K_1_	0.006
		R_2_	0.999
BiVO_4_/zeolite	Pseudo first-order	K_1_	0.003
		R^2^	0.89
	Pseudo second-order	K_1_	0.000
		R_2_	0.999

## 2 Experimental Design, Materials and Methods

### 2.1 Materials and methods

Acid orange 10 dye powder, titanium dioxide (APS: 20 nm and SSA:>200 m^2^.g), Bi(NO_3_)_3_.5H_2_O, NH_4_VO_3_, Na-Y zeolite, sodium hydroxide, and hydrochloric acid were purchased from Sigma Aldridge and Merck companies and were used without further purification. The removal efficiency was calculated by Eq. (1):

(1) $$RE\;\left(\%\right)=\frac{C_{t}-C_{0}}{C_{t}}*100$$

Where *C*_0_ and *C_t_* are the initial and final concentrations of dye at time = 0 and *t*, respectively.

#### 2.1.1 Preparation of BiVO_4_

In a simple and quick method, 0.02 mol of each of Bi(NO_3_)_3_.5H_2_O, and NH_4_VO_3_ were dissolved in 20 mL of 4 M HNO_3_, and 6 M NaOH, respectively, and stirred for 2 h at room temperature. The two solutions were mixed and stirred until a clear yellow solution was obtained. The formed slurry was then transferred to an autoclave for hydrothermal treatment and then was kept at 180 °C for 24 h. After the hydrothermal growth process, the products were washed with distilled water and ethanol and finally placed in an oven at 500 °C for 5 h [1,2].

#### 2.1.2 Preparation of TiO_2_/zeolite and BiVO_4_/zeolite

Here, due to the same synthesis of these two composites, both are explained together. Both composites TiO_2_/zeolite and BiVO_4_/zeolite were synthetized by the hydrothermal method, then they were mixed in equal proportions (50/50) and used in the later applications. The steps were similar to the preparation of BiVO_4_, except for the last step, which was placed in the oven at 400 °C for 2 h.

#### 2.1.3 Nanomaterial experiments

The removal efficiency and photocatalytic oxidation experiments of AO10 solution were studied in a 100 mL pyrex glass vessel as a reactor by the investigated nanomaterials. A 125 W lamp (Philips) enclosed in a quartz casing for TiO_2_/zeolite immersed in the inner part of the reactor and a 12 W LED lamp (white light, light intensity = 28 mW/cm^2^, wavelength emission = 400–600 nm) for BiVO_4_/zeolite located at the top of the reaction vessel were used as light sources. The required reaction was initiated by turning on the LED and UV lamp for two systems and the samples (4 mL) were withdrawn in determined time intervals and filtered by fiberglass filter to separate nanocomposites [3].

### 2.2 Experimental design

In this study, an experimental design software (Design Expert ver. 11.0.1), as well the response surface methodology (RSM) were used to determine the main factors and the interaction between them and square effects, to minimize the number of experiments and save time and cost. RSM is a method dedicated to estimating the relationship between one or more response variables and some independent variables, through a set of designed experiments and regression analysis methods. The effect of initial dye concentration, pH, contact time, and catalyst dosage factors on the dye removal process at five levels was investigated. Analysis of variance (ANOVA) was used to analyze the data. The response variable is presented in the form of a polynomial regression model in Eqs. (2) and (3), for TiO_2_/zeolite and BiVO_4_/zeolite composites, respectively, which are presented as a function of independent variables.

(2) $$\begin{array}{ccc} Y & = & +80.47-10.16*A-7.98*B+13.49*C+1.17*D+0.8094*AB-6.17*AC-2.89*AD\\ & & -\;0.7769*BC-0.8281*BD-2.12*CD-2.98*A^{2}-2.03*B^{2}+6.91*C^{2}-4.95*D^{2}\;\; \end{array}$$

(3) $$\begin{array}{ccc} Y & = & +27.78-6.21*A-1.90*B+8.80*C+4.18*D-0.8862*AB+2.19*AC-2.56*AD\\ & & +\;1.97*BC+0.97*BD+0.4825*CD+4.24*A^{2}-0.5227*B^{2}-0.5290*C^{2}-1.24*D^{2}\;\; \end{array}$$

### 2.3 Adsorption isotherms

The linear diagrams of Langmuir and Freundlich adsorption isotherms for AO10 removal on TiO_2_/zeolite and BiVO_4_/zeolite composites are presented in Figs. 5 and 6, respectively. According to the diagrams and the values of the coefficients obtained in Table 6, it was found that the AO10 dye adsorption on both composites TiO_2_/zeolite and BiVO_4_/zeolite follows the Langmuir model.

### 2.4 Investigation of adsorption kinetics

To investigate the kinetics of AO10 dye adsorption, two kinetic models including pseudo-first order and pseudo-second order kinetic models, were used. The pseudo-second order adsorption kinetics plots for TiO_2_/zeolite and BiVO_4_/zeolite are shown in Figs. 8 and 9, respectively. The coefficients for the kinetic models can be seen in Table 9.

### 2.5 Photocatalytic mechanism of studied composites

Generally, only TiO_2_ and BiVO_4_ can absorb photons and be stimulated to generate electron and holes pairs. In addition, the reaction between holes and OH- and H_2_O absorbed on the surface of the nanostructures particles, results in the production of OH radicals to destroy of AO10 dye. In this process, zeolite as a strong adsorbent can prevent the recombination of electron/hole pairs.

## CRediT Author Statement

**Behzad Rahimi:** Conceptualization, Investigation, Data curation, Software, Resources, Writing - Original Draft, Writing - Review & Editing; **Nayereh Rezaie-Rahimi:** Investigation, Resources, Writing - Original Draft; **Negar Jafari:** Investigation, Resources; **Ali Abdolahnejad:** Investigation, Resources; **Afshin Ebrahimi:** Supervisor, Data curation, Resources, Idea planning, Writing - Review & Editing.

## Declaration of Competing Interest

The authors declare that they have no known competing financial interests or personal relationships that could have appeared to influence the work reported in this paper.
